# Spontaneous renal hemorrhage secondary to choriocarcinoma in a man with congenital hypospadias and cryptorchidism: a case report and literature review

**DOI:** 10.1186/s12885-018-4424-4

**Published:** 2018-05-08

**Authors:** Yi Li, Gang Chen, Han Chen, Shuang Wen, Chao-yu Xiong, Zi-yi Yang, Yun-xiao Zhu, Nathan Jeffreys

**Affiliations:** 1 Department of Urology, The First Affiliated Hospital of Chongqing Medical University, No.1, Youyi Road, Yuzhong District, 400016 Chongqing China; 2 University College London Medical School, Medical School Building, 74 Huntley Street, London, WC1E 6BT England, UK

**Keywords:** Nongestational choriocarcinoma, Male, Spontaneous renal hemorrhage, Hypospadias, Cryptorchidism

## Abstract

**Background:**

Choriocarcinoma is a rare malignant germ-cell tumour, most commonly found in adult women. It infrequently presents as spontaneous renal haemorrhage (SRH). Genital malformation and SRH secondary to choriocarcinoma has previously been only reported in females. We present what we believe to be the first case of a male patient with genital malformation (hypospadias and cryptorchidism) and SRH at presentation of choriocarcinoma.

**Case presentation:**

A 25-year-old man presented to the department with intense pain in the right flank region and lower back. Initial investigations showed spontaneous renal haemorrhage, for which an emergency partial nephrectomy was performed. Clinical, radiological, and pathological investigations suggested a diagnosis of testicular choriocarcinoma with metastases to the right kidney, both lungs, and brain. Initial treatment was with a chemotherapy regimen of cisplatin, etoposide and bleomycin and whole brain radiotherapy; however, 6 months after diagnosis the patient developed liver metastasis, after which time the BEP protocol was switched to ITP with oral apatinib. Despite best efforts, the liver and lung metastasis continued to grow and a decision was made to discontinue active treatment and provide only palliative care until the patient passed away.

**Conclusion:**

Choriocarcinoma is a difficult cancer to diagnose pre-operatively. In male patients with early metastasis, prognosis may be much poorer than in the commoner gestational choriocarcinoma. A multidisciplinary with comprehensive post-surgical intervention is of great importance in the treatment of these patients.

## Background

Primary choriocarcinoma, also called chorionic epithelial carcinoma, is a rare malignant neoplasm that develops in the placental chorionic epithelial tissue niche. It can be divided into two clinical entities – gestational carcinoma and the rarer non-pregnancy carcinoma [1]. All ages and sexes can be affected, although it is rare in adult males. In males, the usual site of the primary lesion is the testis [2] or midline structures, including the mediastinum [3] and intracranium [4]. Spontaneous renal haemorrhage (SRH) at presentation is rare, particularly when coupled with genital malformation, which has previously only been reported in female patients [5, 6]. Here we present the case of a 25-year-old man presenting with SRH secondary to choriocarcinoma, complicated by cryptorchidism and hypospadias. We describe the diagnosis and course of treatment together with a review of the literature.

## Case presentation

**Fig. 1:**
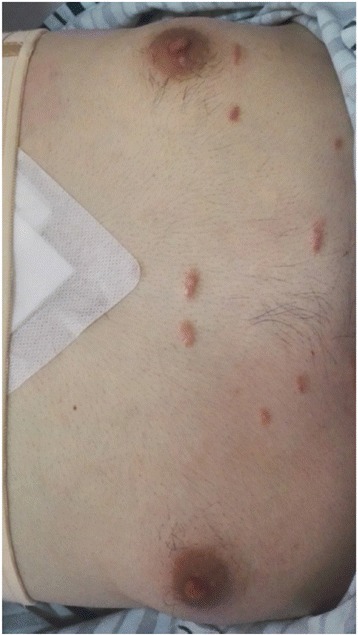
Clinical photograph showing evident gynecomastia

**Fig. 2:**
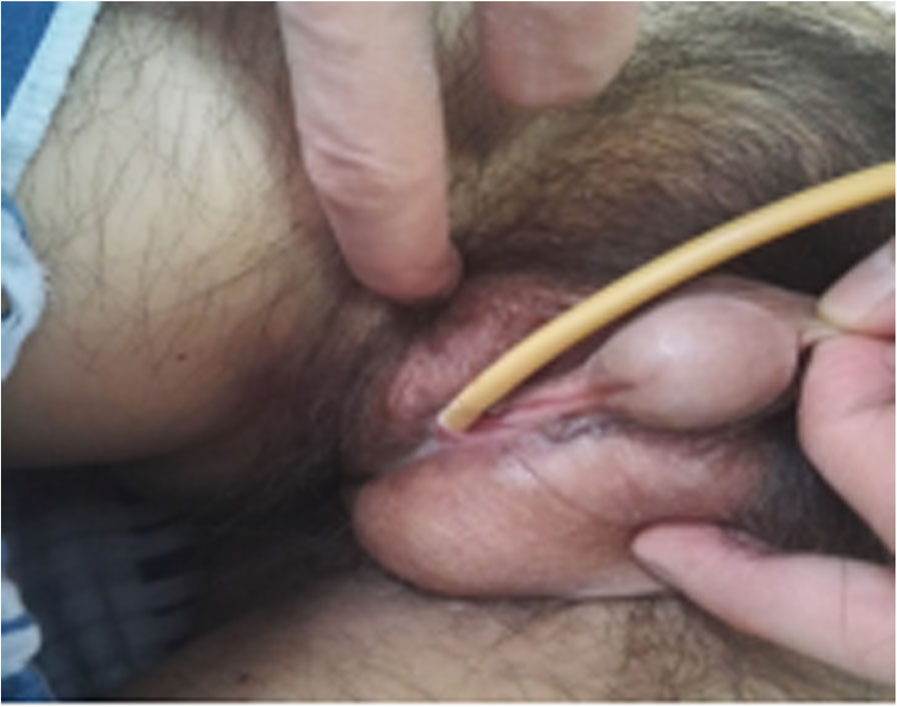
Clinical photograph showing the hypospadias and meatus

A 25-year-old male presented to our hospital with intense pain in the right flank region and lower back. At admission, his blood pressure was 90/60 mmHg and heart rate was 85 bpm. On clinical examination, he appeared anaemic and was cool peripherally. He was noted to have bilateral gynecomastia (Fig. 1) and no obvious Adam’s apple. The abdomen was visibly distended, and there was tenderness to palpation and percussion in the entire right abdominal area. Pain was also present on ballottement of the right kidney. He had hypospadias (Fig. 2) and cryptorchidism, which on questioning had been present since birth in both the patient and his twin brother; neither had yet undergone any corrective procedures for this.

**Fig. 3:**
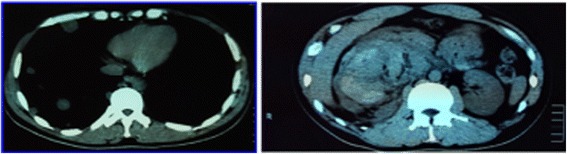
The primary CT

**Fig. 4:**
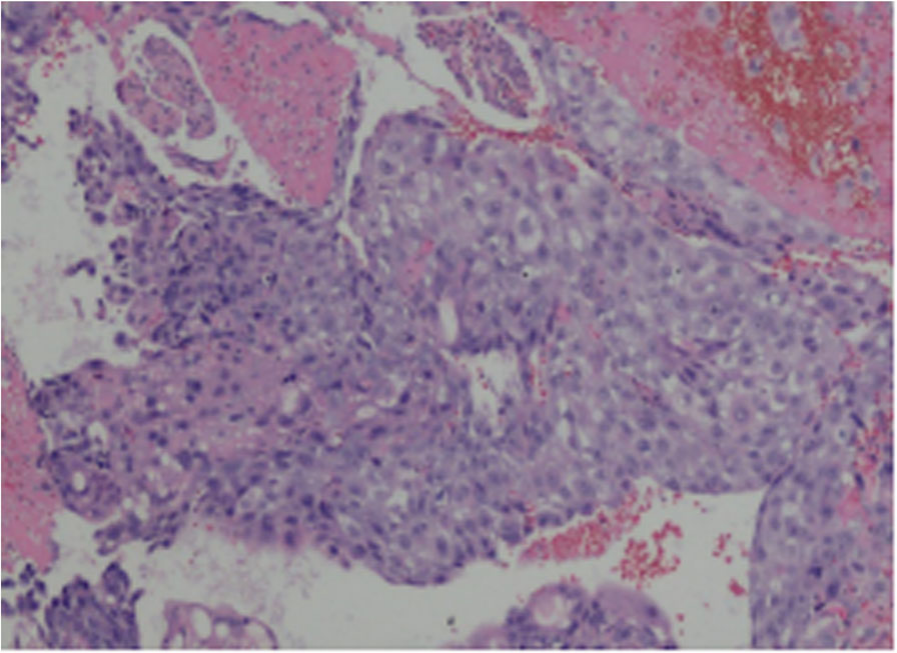
HE stain of kidney biopsy. Necrosis and haemorrhage is visible. Malignant syncytiotrophoblastic cells of different sizes are present. The nuclear membrane is thick, with obvious nucleolus and common nuclear division. Atypia is present

**Fig. 5:**
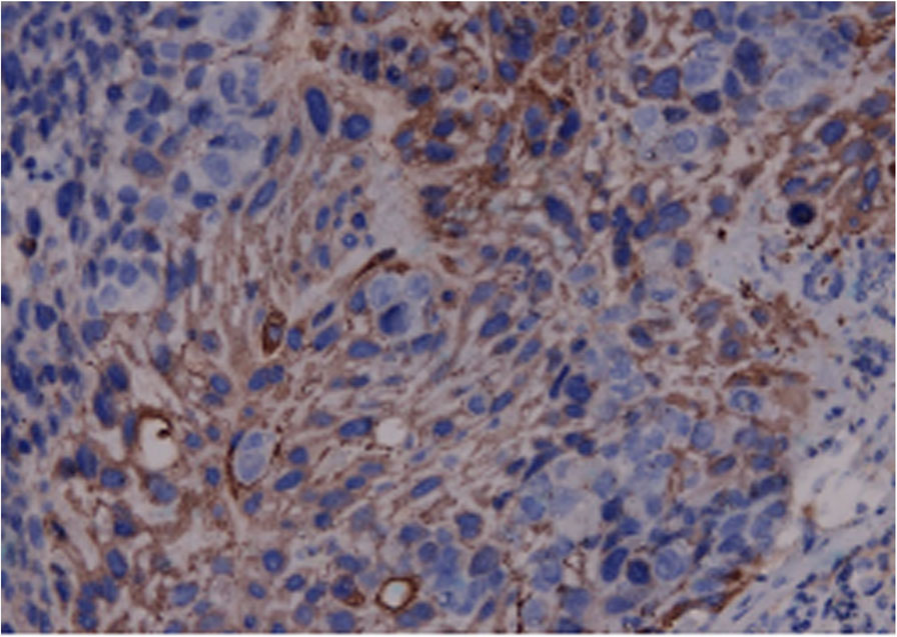
Immunohistochemical test of kidney biopsy showing HCG (+)

Initial blood tests showed an initial Hb concentration of 150 g/L, however three hours after presentation this had dropped to 137 g/L. An ultrasound of the urinary tract showed an enlarged and irregularly-shaped right kidney. CT showed an enlarged right kidney, kidney lesions, and right renal haemorrhage with perirenal hematoma formation (Fig. 3), together with further lesions in the lung. Angiomyolipoma was first considered as this is the most common cause of SRH in our local demographic, the initial management of which is often selective angio-embolization. However, due to the lesions in the kidney and lungs together with genital malformation, we could not rule out a diagnosis of a malignant tumour and therefore, we instead opted to perform an emergency partial nephrectomy to control the haemorrhage, allow early kidney resection and provide histological biopsies. During the operation, a 1000 g retroperitoneal haematoma was found and evacuated. Intraoperatively, the lesions were evident in the middle and lower right kidney, with the lower one having ruptured. Due to the genital abnormalities present and imaging results inconsistent with more common conditions, choriocarcinoma was suspected. Subsequently, post-operative histology samples were taken which confirmed this diagnosis (Fig. 4), with positive immunohistochemistry when stained for β-HCG (Fig. 5).

**Fig. 6:**
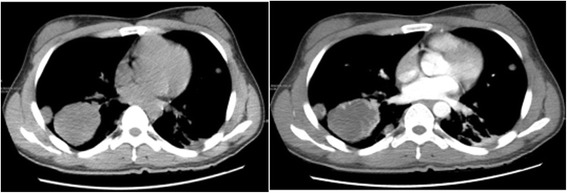
The primary CT showing multiple nodules and masses within the abdomen. The largest measures 5.7 × 6.0 cm

**Fig. 7:**
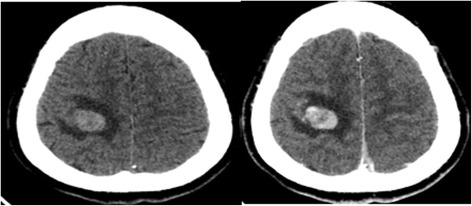
The brain CT scan shows multiple round nodules with peripheral enhancement. The largest is in the right parietal lobe and measures 1.5 cm × 1.9 cm

**Fig. 8:**
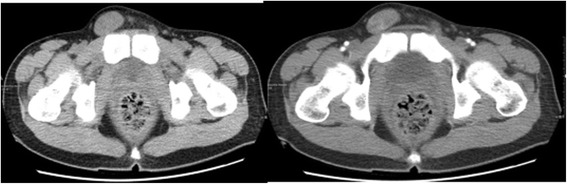
The CT scan showing cryptorchidism with peripheral enhancement in the right groin area

Laboratory investigations showed raised β-HCG, oestradiol, progesterone, Ferritin, and cytokeratin-19 fragment. Post-operative renal function remained within normal limits. Karyotype analysis showed the patient to have a normal 46, XY karyotype. Further CT scans showed additional metastases in the brain and both lungs (Figs. 6 and 7). A contrast CT of the abdomen and pelvis showed epididymal and testicular shadowing in the right groin area; this was edge-enhancing and of uniform density (Fig. 8). At this time, the working diagnosis was right testicular choriocarcinoma with metastases to the right kidney, brain and both lungs.

**Fig. 9:**
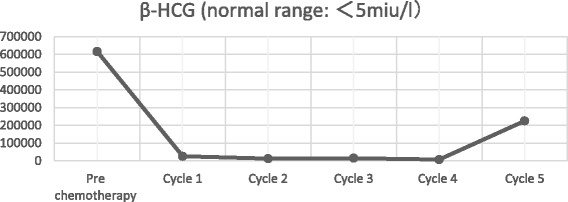
The trend in -HCG levels after each round of chemotherapy

**Fig. 10:**
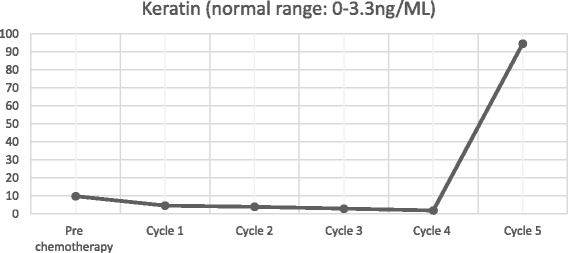
The trend in keratin levels after each round of chemotherapy

**Fig. 11:**
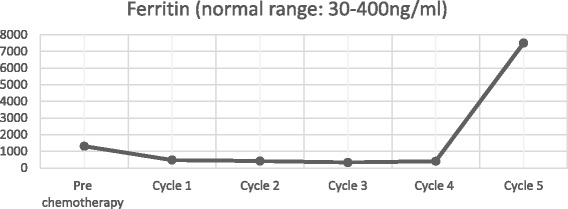
The trend in ferritin levels after each round of chemotherapy

**Fig. 12:**
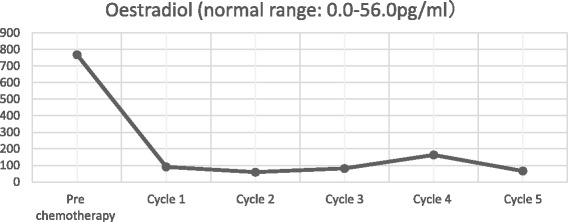
The trend in oestradiol levels after each round of chemotherapy

**Fig. 13:**
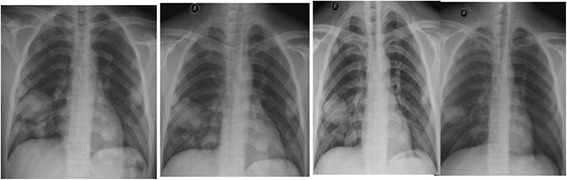
Multiple chest x-rays showing disease progression over time

**Fig. 14:**
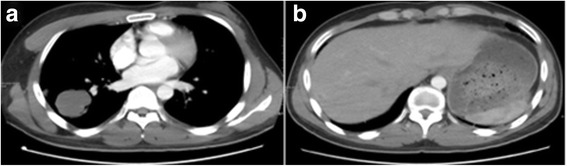
**a** (left): CT showing the initial reduction in size of multiple lesions after BEP chemotherapy. The largest nodule is 4.7 × 4.2 cm. **b** (right): The enhanced CT shows masses of heterogeneous density within the liver parenchyma

**Fig. 15:**
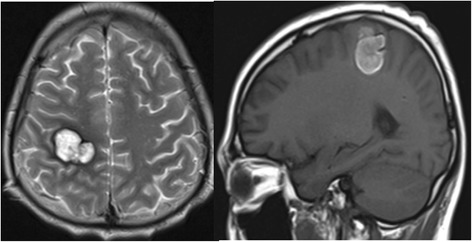
The MRI scans show a reduction in the size of multiple brain metastases within the right Parietal lobe in comparison with the prior images

**Fig. 16:**
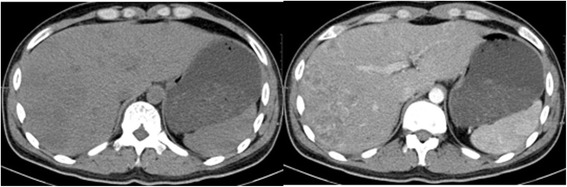
CT showing multiple new masses are present in the liver with peripheral enhancement

**Fig. 17:**
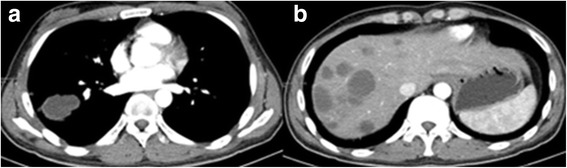
**a** (left): CT showing the decrease in size of the lung lesions in comparison with Fig. 15a. **b** (right): CT showing the increase in size of the liver lesions in comparison with Fig. 17

**Fig. 18:**
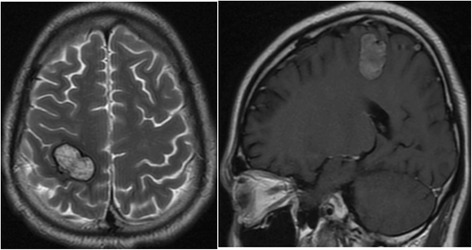
CT showing the decrease in size of right parietal lobe lesions in comparison with Fig. 16

After declining early surgical treatment, the patient was referred to the oncology department for chemotherapy and radiotherapy. His initial treatment included a modified BEP chemotherapy regimen (bleomycin 15,000 IU (d1, d15), etoposide 120 mg (d1 - d5), cisplatin 30 mg (d1 - d5)) with whole brain radiotherapy. Following each round of chemotherapy, the sex hormone levels were monitored (Figs. 9, 10, 11 and 12). Initially, a significant reduction in the blood concentration of oestradiol, keratin and β-HCG was observed. Additionally, chest x-rays, MRI brain and CT scans suggested marked reduction in the size of all metastases (Figs. 13, 14, 15, 16, 17 and 18). However, after the fourth cycle of chemotherapy, multiple liver metastases were found on an enhanced abdominal CT during a work-up of gastritis. The team felt this indicated BEP resistance, and so the BEP regimen was changed to ITP (ifosfamide d1-d5 + taxol d1 + cisplatin d1–4), with the addition of oral apatinib for tumour-targeted therapy.

**Fig. 19:**
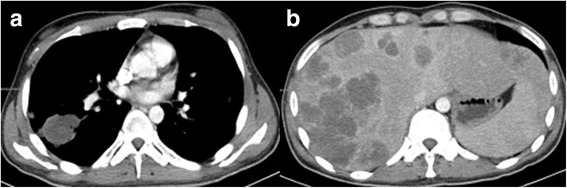
**a** (left): CT showing that the lung lesions present in Fig. 18a have increased in number and size despite ITP therapy. **b** (right): The liver lesions present in Fig. 18b have also increased in number and size

Despite this, one month later enhanced chest and abdominal CT scans showed that several metastases had increased in size (Fig. 19; cf. Fig. 17). Ultrasound showed enlarged lymph nodes in the cervical and axillary groups, with additional calcification in the right inguinal groups. Laboratory investigations showed severe anaemia, hypoalbuminaemia, and a significant rise in β-HCG and other tumour markers. Due to significant side effects and continued disease progression, the patient opted to discontinue active treatment. He later passed away at home.

The patient’s twin brother was also evaluated by the hospital due to the presence of genital malformations on physical examination. These included perineal hypospadias, short penis, chordee, penoscrotal transposition, right cryptorchidism and scrotal splitting. His AFP, β-HCG and other sex hormone levels were within normal limits, and his chromosomal analysis showed the typical male genotype of 46, XY. He opted for a surgical urethral reconstruction, right orchiectomy and correction of the penoscrotal transposition, chordee and scrotal splitting. The pathological specimens taken from his undescended testis showed normal testicular histology.

## Discussion and conclusion

Non-gestational choriocarcinoma, also known as primary choriocarcinoma (PCC), can affect both sexes, with around 1 in 4 cases reported in males. Non-gestational choriocarcinoma is rare, with an incidence ratio of 1:79 when compared to the more common gestational variant of choriocarcinoma. The pathogenesis of PCC is currently unclear, although at present there are three main hypotheses: 1) The tumour stems from primordial germ cells, arising during embryogenesis, which migrate abnormally and so are retained into adulthood [7]; 2) The tumour is a metastasis from a primary gonadal choriocarcinoma [8]; 3) The tumour arises directly in non-gonadal tissues through trophoblastic differentiation, or ‘transdifferentiation’, of some cells [9].

In a review of 113 male patients with PCC, the most common primary site was the testicles (36.2%), followed by the mediastinum, stomach, and liver [7]. Early haematogenous metastases are well documented in both sexes, with PCC preferentially seeding to the lungs (94% of all metastatic choriocarcinoma), vagina (44%), liver (28%) and brain (28%) [10]. Due to this early dissemination, most patients present with symptoms of metastases. In particular, patients can present with haemorrhage, due to PCC preferentially invading the local vasculature. This most commonly occurs in metastases to the lungs, liver and kidneys, and in severe cases can lead to haemorrhagic shock, as in our patient’s initial presentation with SRH.

Due to the wide variety of conditions that can lead to SRH, diagnosis of the underlying cause can be difficult. Despite the most common tumours causing SRH being renal cell carcinoma and angiomyolipoma, it is important to consider metastatic lesions as part of the differential. As in our case, when imaging is not consistent with the more common differentials, other serum tumour markers should be evaluated. In males with cryptorchidism, metastatic choriocarcinoma should be considered and the level of β-HCG measured in order to support or refute this [1]. Importantly, according to the tumour regression hypothesis, the primary tumour may regress early in the disease course and leave behind only metastases, and thus the absence of clinical signs in the testes does not exclude this diagnosis [11, 12]. Furthermore, it has been reported that even in deaths secondary to confirmed metastatic choriocarcinoma, testicular abnormalities can often only be apparent on autopsy.

On physical examination, our patient had gynecomastia, right testicular atrophy and enlargement of the left undescended testicle. Around 50% of patients with non-gestational choriocarcinoma have been reported to have gynaecomastia [13]. The pathophysiology is unclear, but may be the result of a disruption in the serum oestrogen: androgen ratio. The β-HCG produced by the primary tumour can act via a negative feedback pathway to inhibit the secretion of gonadotropin. This in turn decreases the production of androgens. The elevated oestrogen levels can also inhibit testosterone synthetase directly, further reducing the androgen levels. This leads to changes in secondary sexual characteristics, such as the development of gynaecomastia. Therefore, we suggest that gynaecomastia may be an important clinical sign during the initial work-up of SRH that should prompt investigation into this malignancy as a potential differential.

As previously discussed, pre-operative diagnosis of PCC can be difficult as patients often present with symptoms secondary to the effects of metastatic lesions. This makes finding the primary tumour site difficult, particularly as abnormalities on clinical examination can be absent. β-HCG is produced by non-gestational choriocarcinoma, and serum levels are elevated in 96.4% of patients with this tumour [2]. Shinoda et al [14]. suggests that patients with very high β-HCG (> 1000 miu/ml) can be diagnosed with choriocarcinoma even before confirmatory biopsy specimens. Our case supports this, as the initial β-HCG in our patient was much higher than this level, at 616,365miu/ml. This indicates the importance of serum β-HCG levels in the initial work-up prior to biopsy.

Due to the rarity of non-gestational choriocarcinoma, most treatment options are empirical. Usually, surgical resection of the primary tumour and metastases is combined with chemotherapy in order to improve prognosis, particularly for early stage cancers [7]. In males, four cycles of the BEP (bleomycin, etoposide and cisplatin) regimen is often used [15]. As in our case, response may be poorer in patients with advanced disease at presentation, as the optimum window for chemotherapy is early is the disease course. Additionally, the blood-testis barrier provides a physiological barrier which prevents chemotherapy agents from acting at the primary tumour site. Our patient refused surgery to remove the remaining enlarged testicle, which we hypothesise may be in part responsible for the later disease recurrence and metastatic spread to the liver.

It is important to consider why neither the patient nor his twin brother had presented sooner, as the genital abnormalities noted were evident from birth. We suggest this is the result of two main factors: 1) the poor educational background of this family, leading to ignorance of the treatment options available; and 2) the societal taboo of discussing and treating conditions relating to the genital area. Therefore, we suggest it is necessary to address these issues to improve outcomes for this condition.

In summary, non-gestational choriocarcinoma is a rare cancer in men, and often presents at an advanced stage with poor prognosis. Clinicians should be aware of the typical clinical findings such as testicular atrophy, and gynaecomastia which would suggest this diagnosis in the evaluation of metastases of unknown origin. Serum β-HCG is a sensitive laboratory test during the initial work-up. Treatment options usually include complete resection of the primary site, with adjuvant chemotherapy to improve prognosis. Control of metastatic disease is difficult and dependent on the number and location of metastases; this can include both surgical and chemotherapeutic options.

## Abbreviations

BEP: Bleomycin 15,000 IU (d1, d15), Etoposide 120 mg (d1 - d5), Cisplatin 30 mg (d1 - d5))
CT: Computed tomography
HCG: Human chorionic gonadotropin
ITP: Ifosfamide d1-d5, Ttaxol d1, Cisplatin d1–4
MRI: Magnetic resonance imaging
PCC: Primary choriocarcinoma
SRH: Spontaneous renal hemorrhage
